# Iron Bioavailability from Ferrous Ammonium Phosphate, Ferrous Sulfate, and Ferric Pyrophosphate in an Instant Milk Drink—A Stable Isotope Study in Children

**DOI:** 10.3390/nu14081640

**Published:** 2022-04-14

**Authors:** Richard F. Hurrell, Trinidad P. Trinidad, Aida C. Mallillin, Rosario S. Sagum, Jasmin Tajeri Foman, Qiaoji Li, Christophe Zeder, Peter Kastenmayer, Andreas Rytz, Magalie Sabatier, Ines Egli

**Affiliations:** 1 Laboratory of Human Nutrition, Institute of Food, Nutrition and Health, ETH Zurich, 8092 Zurich, Switzerland; richard.hurrell@hest.ethz.ch (R.F.H.); jasmin.tajerifoman@rdls.nestle.com (J.T.F.); christophe.zeder@hest.ethz.ch (C.Z.); ines.egli@ethrat.ch (I.E.); 2 Department of Science and Technology, Food and Nutrition Research Institute, Taguig City 1630, Philippines; mallillin_aida@yahoo.com (A.C.M.); rssagum@ust.edu.ph (R.S.S.); 3 Clinical Research Unit, Nestlé Research, Société des Produits Nestlé S.A., Vers-Chez-Les-Blanc, 1000 Lausanne, Switzerland; andreas.rytz@rdls.nestle.com; 4 Nestlé Research and Development China Ltd., Building 5, No 6 Jiu Xian Qiao Road, Chao Yang District, Beijing 100102, China; qiaoji.li@rd.nestle.com; 5 Nestlé Institute of Health Sciences, Nestlé Research, Société des Produits Nestlé S.A., Vers-Chez-Les-Blanc, 1000 Lausanne, Switzerland; pkastenmayer@yahoo.com

**Keywords:** ferrous sulfate, ferrous ammonium phosphate, ferric pyrophosphate, stable isotope, absorption, milk, children

## Abstract

Ferrous ammonium phosphate (FAP) is an iron salt that has been developed for the fortification of food matrices sensitive to color and flavor changes. The objective of the study was to measure iron absorption from FAP in young children and compare it to a previous evaluation of FAP in young women. A double-blind randomized crossover study with two parallel arms was used to evaluate the iron absorption from FAP added to reconstituted milk powder in comparison to that from ferrous sulfate (FeSO_4_) and ferric pyrophosphate (FePP). Iron absorption was measured in 39 children aged 3- to 6-years-old using erythrocyte incorporation of stable Fe isotopes (^57^Fe, ^58^Fe). The geometric mean iron absorption in iron replete children from FAP, FeSO_4_ and FePP from milk was 8.3%, 7.6% and 2.1%, respectively. Iron absorption from FAP and FeSO_4_ fortified milk was not significantly different (*p* = 0.199); however, it was significantly higher than from FePP fortified milk (*p* < 0.001). Iron bioavailability from FAP and FePP relative to FeSO_4_ (relative bioavailability (RBV)) was 110% and 33%, respectively. The RBV of FAP (110%) in iron replete children was higher than previously reported RBV (71%) in mainly iron deficient women. The difference in iron status between the children and women in the respective studies may explain the different RBV values and is discussed.

## 1. Introduction

Anemia affects a third of the world’s population [1]. Based on 2011 global estimates, 43% of preschool children and 33% of nonpregnant women were anemic, with the highest burden in Africa and South Asia [2]. The etiology of anemia is varied and complex with iron deficiency (ID), inflammation, hemoglobinopathies, and hookworm being important causes [1,3]. Although ID is the main driver in high-income countries, in low- and middle-income counties, especially those in sub–Saharan Africa with widespread infections, inflammation may be the major cause of anemia. Recent estimates suggest that only 25% and 37% of anemia in, respectively, preschool children and women of reproductive age living in countries with widespread infections and inflammation is associated with ID [4]. Iron is essential for hemoglobin synthesis, for a range of key enzymes essential for normal brain development in the fetus and the child, for optimum immune defense, and for efficient energy production [5].

Food fortification with iron is generally regarded as the most cost effective and sustainable long-term approach for decreasing the prevalence of ID [6], and iron compounds used for food fortification must be carefully selected with respect to bioavailability and their potential to cause unacceptable sensory changes to the food product. For a given food product, the iron compound chosen is that which has the highest bioavailability without provoking unacceptable color, flavor, odor, or texture changes in the food during the production, storage, or preparation for consumption. In relation to milk powders, this includes changes after reconstitution with water.

It is generally accepted that more water-soluble iron salts have a higher bioavailability, but also more potential to cause unacceptable sensory changes in the product. On the other hand, the more insoluble compounds create fewer sensory problems but are often less well absorbed. Water soluble iron compounds, such as ferrous sulphate (FeSO_4_), the reference salt for iron absorption [7], frequently cause the most adverse sensory changes in sensitive foods. This is because free, solubilized iron has a distinct metallic taste; it can form unacceptable colored complexes with polyphenol compounds in fruits and vegetables, and can oxidize fats in lipid-containing foods such as wheat flour, whole milk or full cream milk powders [6]. Water insoluble compounds, on the other hand, cause no or few sensory changes but they may be less well absorbed as they may not dissolve completely in the dilute acid of gastric juice during digestion.

Whole milk powders are commonly used as a fortified food source to provide additional iron to children older than 3 years. Ferric pyrophosphate (FePP) is widely used to fortify milk powders targeted at older children. It is insoluble in water and causes few if any sensory changes to foods. After processing and reconstitution with water, the FePP remains suspended in the milk drink attached to the other milk constituents. However, due to its poor solubility at gastric pH, iron absorption from FePP is low [8,9,10] and reported to be about 30% compared to the well absorbed FeSO_4_ in milk [11]. Another iron compound widely used to fortify foods sensitive to sensory changes [6,12,13,14,15] is ferrous fumarate. This compound is poorly soluble in water and highly soluble in gastric juice, as indicated by reports showing that ferrous fumarate has a similar fractional iron absorption to FeSO_4_ in iron replete infants, school children and women [12,13,14]. However, it has a deep red color and is not a suitable fortificant for whole milk powder [16].

Ferrous ammonium phosphate (FAP) is light green and was developed for the fortification of milk powder. It has a molecular weight of 168.85 g/mol, contains approximately 30% iron (*w*/*w*), and its approximate cost per mg iron relative to FeSO_4_ and FePP is 6.0 and 1.7, respectively [11]. It is much more soluble in dilute acid than FePP e.g., 57.8% vs. 11.6% at pH 1.7 [11], but somewhat less soluble than ferrous fumarate (i.e., 100% at pH 2) [17]. It has been classed as generally regarded as safe (GRAS) for the general population aged over 3 years by the Joint FAO/WHO Expert Committee on Food Additives (JECFA) [18] and by the European Food Safety Authority [19].

The relative absorption of FAP was first measured in young women consuming a full cream instant milk drink fortified with FAP and compared to that of FePP and FeSO_4_ [11]. The authors reported significantly lower iron absorption from both FAP and FePP than from FeSO_4_ (7.4% and 3.3%, respectively, vs. 10.4%, *p* = 0.001, corresponding to a relative iron bioavailability to FeSO_4_ (RBV) of 71 and 33%); yet iron absorption from FAP was significantly better than from FePP (*p* < 0.0001). As the target consumer group for fortified full cream milk powder are children, the study has been repeated in this population. The objective of this trial was to compare the fractional iron absorption in children aged 3–6 years from FAP added to a reconstituted whole milk powder with that of FeSO_4_ and FePP. Iron absorption was measured using the erythrocyte incorporation of stable isotopic labels 14 days after the consumption of the isotopically labeled test drinks.

## 2. Materials and Methods

### 2.1. Subjects

A total of 40 apparently healthy children were recruited at the Kindergarten (Bicutan Elementary School) in Metro Manila, Philippines by the Food and Nutrition Research Institute (FNRI). Forty children fulfilling the inclusion criteria, i.e., age 3–6 years (inclusive), a normal BMI for age according to the WHO standard, non-anemic according to the WHO cutoff, apparently healthy, no intake of medication or vitamin or mineral supplements 2 weeks before the start of the study and during the study, no participation in another clinical trial, and the ability to comply with the study protocol, were included. The study was conducted at the FNRI in November and December 2009 according to the guidelines laid down in the Declaration of Helsinki. The study protocol was approved by the FNRI Institutional Ethics Review Committee, Manila, Philippines and the ETH Ethics Committee, Zurich, Switzerland, and written informed consent was obtained from all parents of the participating children. The study was conducted according to GCP guidelines.

### 2.2. Study Design

A double-blind randomized crossover study with two parallel arms was applied to limit the duration of the study. Within each arm, the subjects crossed over on two treatments, i.e., FeSO_4_ vs. FAP (A vs. B) in the first arm, and FeSO_4_ vs. FePP (A vs. C) in the second one over 4 days. In arm 1, 20 subjects were randomly assigned to start the drink consumption by the sequence ABAB or BABA. In the second arm, 10 subjects were randomly assigned to the sequence ACAC, and 10 others to CACA. The sample size was established assuming the comparable effect size and variability as observed in adults [11], with two-sided alpha = 5% and power = 80%.

Venous blood samples were drawn after an overnight fast for the determination of the iron status parameters, i.e., hemoglobin (Hb), ferritin, and C-reactive protein (CRP) as an inflammation marker, in the week preceding the test meal administration. The body weight and height were measured. On day 1, the first labeled test meal was administered after an overnight fast. The following day (day 2), the second test meal was administered according to the same procedure, as well as on days 3 and 4. There was no washout period between the 4 days of labelled test drink consumption to be close to the pattern of milk consumption in this age group, i.e., one serving of milk per day. A second venous blood sample was drawn 14 days after the consumption of the last test drink (day 18). The study design is provided in Supplementary Material Figure S1.

### 2.3. Isotopic Labels

Isotopically labeled FeSO_4_ (^58^Fe) and FAP (^57^Fe) and FePP (^57^Fe) were prepared by Dr. Paul Lohmann GmbH (Emmerthal, Germany) from isotopically enriched elemental iron (Chemgas, Boulogne, France) using a lab scale procedure that follows closely the procedures employed for the production of commercially available products. The iron content of the compounds was assessed via isotope dilution mass spectrometry using a gravimetric standard prepared from a certified iron isotopic reference material (IRM-014, EU JRC Institute of Reference Material and Measurements, Geel, Belgium). The isotopic enrichment of ^58^Fe as FeSO_4_ was 99.5%, ^57^Fe as FAP was 97.5% and ^57^Fe as FePP was 97.9%. The isotopically labeled compounds were weighed into vials at the Human Nutrition Laboratory (HNL), ETH Zurich, transported to the study site, and shortly before consumption the respective vial was emptied into the test meal. The exact amount of the compound was determined by weighing the vial before and after emptying. A straw was used to mix the isotopically labeled compound with the test drink and the straw was used to consume the test drink. The emptied glasses were rinsed twice with 20 mL water and the washings were consumed to ensure the complete intake of the isotopically labeled compounds and test drink.

### 2.4. Test Drink

The test drinks consisted of full cream milk powder produced at Nestlé Product Technology Center, Konolfingen, Switzerland and reconstituted with purified water. The milk powder was produced according to the specifications for a commercial milk powder but without added iron. The milk powder (26 g) was weighed into a plastic glass; then, 180 g water was added and mixed with a plastic spoon. The milk drink contained 6.5 g protein, 7.4 g total fat, 9.7 g carbohydrates, and 22.6 mg ascorbic acid. Isotopically labeled iron compounds were added, as described above, at the level of 2 mg iron per test drink, thus corresponding to a fortification level of 10 mg iron per liter of prepared milk.

### 2.5. Test Drink Administration

The subjects were reminded to refrain from the intake of foods (after 8 pm) and drinks (after midnight) until the administration of the test drinks on the following morning. On day 1, the first labeled test drink was administered after an overnight fast and the following day (day 2), the second test meal was administered according to the same procedure. This was repeated on days 3 and 4. The test drinks were fed under strictly standardized conditions under close supervision of the investigators. No intake of food and fluids was allowed 3 h after the feeding and the children were under supervision at FNRI during this time. Compliance with the protocol was monitored by questioning the subjects on the following visit.

### 2.6. Blood Sampling and Analysis

Blood samples were drawn by experienced medical technologists using EDTA coated vacutainers. The first blood sample was drawn in the week preceding the test drink administration to determine the iron status and a second blood sample 14 days after administration of the last test drink for isotopic analysis. Hb was measured in whole blood on the day of collection using the cyanmethemoglobin method. Plasma was separated for ferritin and CRP analysis using a radioimmuno and immuno-turbidimetric assay, respectively. These measurements were performed at FNRI and a service laboratory in Manila. Anemia was defined as Hb < 110 g/L for children <60 months and Hb < 115 g/L for children 60–72 months. Iron deficiency was defined as ferritin <15 µg/L [6]. The normal range for CRP was 0.1–2.8 mg/L. Isotopically enriched blood samples were analyzed in duplicate for iron isotopic composition using chemical blank monitoring. Whole blood samples were mineralized by microwave assisted digestion (MLS Ethos, MLS, Leutkirch, Germany) using a mixture of HNO_3_ and H_2_O_2_, which was followed by the separation of the sample iron from the matrix by anion-exchange chromatography and a solvent-solvent extraction step into diethylether [20]. All isotopic analyses were performed at HNL by negative thermal ionization mass spectrometry (NTI-MS) using a magnetic sector field mass spectrometer (MAT 262, Thermo-Finnigan, Bremen, Germany) equipped with a multi-collector system for the simultaneous detection of generated FeF_4_^−^ ions [20].

### 2.7. Calculation of Iron Absorption

Based on the shift of the iron isotope ratios in the blood samples and the amount of iron circulating in the body, the amounts of ^57^Fe and ^58^Fe isotopic label present in the blood 14 days after the last test meal administrations was calculated based on the principles of isotope dilution and considering that the iron isotopic labels are not monoisotopic [21]. The circulating iron was calculated based on blood volume and Hb concentration [22]. The blood volume calculations were based on the body weight and height [23]. For the calculations of fractional absorption, 90% incorporation of the absorbed iron into red blood cells was assumed.

### 2.8. Food Analysis

The iron and calcium content of the milk powder were determined in triplicate analysis, respectively, by graphite furnace and flame atomic absorption spectrometry (AA240, Agilent Technologies, Santa Clara, CA, USA), after microwave-assisted mineralization in a mixture of HNO_3_ and H_2_O_2_ (MLS Ethos).

### 2.9. Statistical Analysis

The iron absorption was log10-transformed to achieve normality. Log10 iron absorption was analyzed by a mixed model that contained the sequence and treatment (FePP, FAP, FeSO_4_) as fixed effects and the subject as a random effect. The mixed model modeled the two crossover arms jointly. The treatment differences for FeSO_4_ vs. FAP and FeSO_4_ vs. FePP were estimated within the subjects, and the treatment difference for FAP vs. FePP was estimated between the subjects. The two treatment differences were estimated from the mixed model via appropriate contrasts. Additionally, the *p* values were adjusted for multiple tests. Thus, the experiment-wise false-positive rate was controlled at a 5% level. The model-based estimates of the log10(x) mean absorption rates were back-transformed and called geometric means. An analysis was performed with R (Version 2.6.1, R Foundation for Statistical Computing, Vienna, Austria) using the libraries “nlme” and “multcomp”.

## 3. Results

### 3.1. Subjects

Twenty children were included in each arm; one subject from arm 2 was excluded after the second visit due to increased body temperature. Thus, 20 and 19 subjects completed the study in arm 1 and 2, respectively. The subjects’ characteristics and iron status are presented in Table 1. There was no significant difference between the two groups for any of the indicators. None of the subjects was anemic or iron deficient. Elevated CRP values were detected in 8 children of the 40 selected for the study during the screening (i.e., CRP > 2.8 mg/L; 3 subjects in arm 1 and 5 subjects in arm 2), and they were not retested on the days of feeding. However, none of the children (except one excluded at the 2nd visit) had signs of infections as determined by body temperature on the days of feeding.

### 3.2. Tested Drink

The native iron and calcium content of the test drinks were 56.2 ± 0.5 µg and 245 ± 7 mg per 200 mL serving, respectively. The average amount of added labelled iron as FeSO_4_, FAP and FePP was 1.9 ± 0.24 mg per 200 mL serving. The calculated molar ratio of ascorbic acid to iron was 3.5:1.

### 3.3. Iron Absorption

The results of iron absorption (geometric mean (−SD; +SD) from the fortified milk are shown in Table 2. The iron absorption from FAP was 8.3% (4.36, 15.84) and slightly, but not significantly higher than from the milk fortified with FeSO_4_, which was 7.6% (3.93, 14.68) (*p* = 0.199). In arm 2, the geometric mean iron absorption from the milk fortified with FePP was low at 2.1% (1.08, 4.05), while the absorption from the same drink fortified with FeSO_4_ was significantly higher at 6.24% (3.25, 11.96) (*p* < 0.001). The relative iron absorption to FeSO_4_ (RBV) from FAP and FePP were 110% and 33%, respectively. A comparison between the arms showed no difference for FeSO_4_ (*p* = 0.358); however, the iron absorption from FAP was significantly higher than that from FePP (*p* < 0.001).

The effect of plasma ferritin on iron absorption was investigated using mixed models. The slopes were found to be not significantly different from 0 and were not influenced by the salt type, neither in arm 1 (*p* = 0.911) nor in arm 2 (*p* = 0.838).

## 4. Discussion

This study is the first study to evaluate iron absorption from FAP in children. It demonstrated that the fractional iron absorption by young children from FAP added to reconstituted whole milk powder is similar to that of FeSO_4_, and 3-fold higher than FePP. The result, however, was somewhat unexpected as, in the earlier study, iron absorption from FAP in young women was only 71% of that from FeSO_4_ (*p* < 0.0002), as showed in Table 3.

One possible reason for this difference in the RBV between the two studies is that the subjects in the previous FAP study [11] were women with low iron status (mean serum ferritin 18 µg/L, 7 subjects out of 19 with iron deficiency), while the children in the present FAP study were all iron replete (mean serum ferritin 59 µg/L, no iron deficiency). Evidence to support this hypothesis comes from reports that other poorly water-soluble iron compounds, such as ferrous fumarate and FePP, give much lower RBV values in subjects with ID than they do in iron replete subjects. For instance, in infants, young children (2–5 yo), and adult women consuming a milk–maize drink, iron from ferrous fumarate was absorbed to the same extent as from FeSO_4_ (the RBV was close to 100% in all population groups) [12]. This contrasts with results from studies made in Mexico [24] and Bangladesh [25], which indeed showed that iron absorption from ferrous fumarate, in mostly iron deficient young children, was only 30% of that from FeSO_4_ (RBV = 30). Differences in iron status have also been reported to influence the RBV of FePP in adults [7,26,27], with RBVs in single subjects calculated to be as low as 15% in a women with ID (serum ferritin < 5 µg /L) and approaching 100% for women with adequate iron status (serum ferritin >35 µg /L ) [7]. The explanation given for this observation with FePP, was that iron deficient subjects upregulated iron absorption more efficiently from FeSO_4_, a compound that readily dissolves in the gastric juice during digestion, than from FePP, a compound that only poorly dissolves in the gastric juice during digestion [26,27]. It is not clear whether the same explanation could explain the low RBV values for ferrous fumarate reported in iron deficient children, as ferrous fumarate would be expected to be close to a complete dissolution during digestion, in both iron replete and iron deficient subjects, but the iron would dissolve at a much slower rate. It is possible, therefore, that the rate of dissolution of the iron compound in the gastric juice also influences the efficiency of upregulating iron absorption in ID. Another explanation could be the efficiency of gastric acid dissolution of ferrous fumarate was somehow diminished in the malnourished, young, iron deficient children.

When the body upregulates iron absorption because of iron deficiency, the lower iron concentrations in the intestinal cells from these less soluble iron compounds may not be able to match the rate of iron transfer from the enterocyte into the plasma that is achieved when FeSO_4_ is used for iron fortification. In subjects with normal iron status, when iron absorption is not upregulated, the iron concentration in the enterocytes originating from ferrous fumarate is sufficient to be transferred into the plasma at the same rate as FeSO_4_. FAP is more soluble in dilute acid than FePP, but less soluble than ferrous fumarate, so would also be expected to give lower RBV values in iron deficient subjects, explaining the lower RBV of FAP reported earlier in the women with ID [11]. Consequently, it is probable that the absorption of iron from FAP in iron deficient children would also be significantly lower than that of FeSO_4_. It should be noted that the RBV values reported are obtained from a single meal absorption study and that, over long-term consumption of a fortified food, the children would gradually recover from iron deficiency. At this stage, the absorption value of FAP (and ferrous fumarate) would likely be similar to that of FeSO_4_.

Milk powders and milk products are frequently used as vehicles for iron fortification, as well as vehicles for the provision of vitamins and other trace elements to populations at risk of micronutrient deficiencies. In relation to iron fortification, commercially fortified whole milk powder targeted at children >3 years is usually fortified with FePP, so as to obtain the optimum sensory properties, and ascorbic acid is added to improve iron absorption. Milk contains two components that can impair iron absorption. These are calcium [28] and proteins (casein and whey when evaluated from a semi-synthetic liquid meal) [29]. Their impact on iron absorption is reported to be much less strong than phytic acid or polyphenols and their modest inhibition in milk products is adequately overcome by the addition of ascorbic acid [30,31]. The WHO recommends that ascorbic acid be added with iron at a 2:1 molar ratio in milk matrices [6]. Ratios lower than this have resulted in very low fractional iron absorption from FePP added to milk-based products, even in mildly anemic children [10].

Our study indicates that FAP would be a much better iron compound than FePP for the fortification of whole dried milk powders targeted at children >3 years. Iron absorption from FAP by iron replete children was about 8% and, with an iron concentration of 10 mg per liter, a serving of 200 mL fortified milk would provide about 160 µg of absorbed iron. The daily iron requirement for absorbed iron in children aged 4–6 years is 0.5 mg per day [32], and a serving of FAP fortified milk co-fortified with ascorbic acid would cover about 30% of this requirement. Conversely, taking 2.1% as the iron absorption from FePP, a serving of FePP fortified milk would only cover about 8% of the -requirement for absorbed iron in this age group. Such a low contribution may be part of the explanation for the significant but still marginal effect of the fortified milk and cereals reported in children >5 years old in decreasing iron deficiency anemia when FePP is used [33]. A way to compensate for the low FePP bioavailability would be to increase the level of fortification as advised by the WHO [6,34]. Although iron deficient children would likely upregulate iron absorption from FAP to a lesser extent than FeSO_4_, fractional iron absorption from FAP fortified reconstituted milk powder is likely be at least similar, if not higher, than that reported in the present study. Regular consumption would, thus, gradually correct iron deficiency, and then the iron fortified milk would help to maintain an adequate iron status.

An alternative compound that could be used to fortify whole milk powders is ferrous bisglycinate. This iron chelate is reported to not dissociate, or to partially dissociate, in dilute acid. Until now, ferrous bisglycinate (without the addition of ascorbic acid) has been the only iron compound used to fortify liquid milk. This chelate overcomes, to some extent, the inhibitors of iron absorption in milk and is better absorbed than FeSO_4_ (in the absence of ascorbic acid). It is reported to be used in the National Costa Rican fortification program for liquid milk and powdered milk (i.e., a level of 1.4 mg iron/serving) [35,36]. Its wider use, however, is prevented by its high cost and potential to cause sensory changes at higher dose and/or in some sensitive foods [37]. Another promising recent approach is the development of a soluble casein-iron-phosphate complex, which can be added to milk with no unacceptable sensory changes. This compound, described as ferric phosphate clusters stabilized in solution by casein molecules, has been reported to be as well absorbed as FeSO_4_ from milk both in vitro and in isotopic studies in human subjects [38,39]. As with FeSO_4_, the casein-iron-phosphate complex would require the addition of ascorbic acid to ensure an optimal absorption.

Our study has several strengths: it is the first study evaluating iron absorption from FAP in milk in children, a target group that consumes fortified milk; and the use of stable isotopes and intrinsic labelling has allowed sensitive and specific iron measurement. A limitation of the study is that the RBVs of FAP and FePP to FeSO_4_ were measured in two separate study arms to keep the same design as in the previous study in women, while the absorption of the three iron salts could have been measured in a same group using ^54^Fe. Nevertheless, as the iron status of the subjects in the two arms was not significantly different, the comparison of the iron absorption from FAP and FePP was possible without a normalization of the data for this parameter.

In conclusion, the iron from milk fortified with FAP and ascorbic acid was well absorbed. The fractional iron absorption from FAP in iron replete children was similar to that from the soluble FeSO_4_ reference compound. The RBV of FAP (110%) in children of good iron status was higher than the RBV (71%) previously reported in women, with iron deficiency. The difference in iron status between the children and the women in the respective studies is suggested to be the reason for the different RBV values.

## Figures and Tables

**Table 1:** Subjects’ characteristics for the 2 study arms (Mean ± SD, unless otherwise stated).

	Arm 1	Arm 2
Number of subjects (n)	20	19
Age (month)	69.7 ± 3.4	68.1 ± 3.2
Gender ^1^	6F-14M	7F-12M
Weight (kg)	18.2 ± 1.5	17.7 ± 2.2
Height (cm)	109.3 ± 4.1	109.3 ± 4.5
BMI (kg/cm^2^)	15.2 ± 0.6	14.8 ± 0.9
Hemoglobin (g/L)	130.2 ± 5.5	127.9 ± 8.8
Plasma ferritin (µg/L) ^2^	59 (33; 107)	57 (37; 90)
CRP (mg/L) ^2^	0.89 (0.32; 2.44)	0.70 (0.22; 5.71)

^1^ F = female, M = male; ^2^ Geometric mean (−SD; +SD); CRP = C-reactive protein.

**Table 2:** Iron absorption in children, descriptive statistics (Geometric mean % −1SD; +1SD, and log10 (Mean ± SD)) per treatment, and comparison in each study arm, as well as the estimated effect between the study arms.

		Geometric Mean% (−SD; +SD)	Log10 (Mean) (log% ± SD)	*p*-Value
Arm 1	FAP	8.31 (4.36, 15.84)	0.92 ± 0.28	
	FeSO_4_	7.58 (3.93, 14.68)	0.88 ± 0.29	
	FAP-FeSO_4_	+0.73	+0.04 ± 0.11	0.199
Arm 2	FePP	2.09 (1.08, 4.05)	0.32 ± 0.29	
	FeSO_4_	6.24 (3.25, 11.96)	0.79 ± 0.28	
	FePP-FeSO_4_	−4.15	−0.48 ± 0.14	<0.001
FAP-FePP	(FAP-FeSO_4_)			
	−(FePP-FeSO_4_)	+4.88	+0.52	<0.001

**Table 3:** Relative iron bioavailability (RBV) for ferrous ammonium phosphate (FAP) and ferric pyrophosphate (FePP), to that from ferrous sulfate (FeSO_4_), in reconstituted milk determined using the erythrocyte stable isotope incorporation technique.

Subjects	n	Plasma Ferritin Concentration	Compound	Fe Dose (mg)	AA	RBV%	Ref.
Young women	19	17.8 µg/L	FAP	2.5 ^1^	✓	71 *	[11]
	19	16.8 µg/L	FePP	2.5	✓	32	
Children	20	59 µg/L	FAP	2 ^2^	✓	110	Present study
	19	57 µg/L	FePP	2	✓	33	

* Significantly different than 100%; AA: Ascorbic acid.^1^ in 250 ml serving size of milk; ^2^ in 200 serving size of milk.

## Data Availability

The data presented in this study are available on request from the corresponding author. The data are not publicly available due to privacy.

## Supplementary Materials

The following are available online at https://www.mdpi.com/article/10.3390/nu14081640/s1, Figure S1: Study design. Stable isotopes were administered over 4 alternative days with the test drink (milk), i.e., A: ferrous sulfate labelled with ^58^Fe; B: ferrous ammonium phosphate labelled with^57^ Fe; C ferric pyrophosphate labelled with ^57^Fe, after the subjects had fasted overnight.
