# Supplementary material for: Iron Bioavailability from Ferrous Ammonium Phosphate, Ferrous Sulfate, and Ferric Pyrophosphate in an Instant Milk Drink—A Stable Isotope Study in Children
Source: Nutrients. 2022 Apr 14;14(8):1640. doi: 10.3390/nu14081640 (PMC9031871; doi:10.3390/nu14081640)
Supplement: Supplementary file 1 [file nutrients-14-01640-s001.zip › nutrients-1676987-supplementary.pdf]

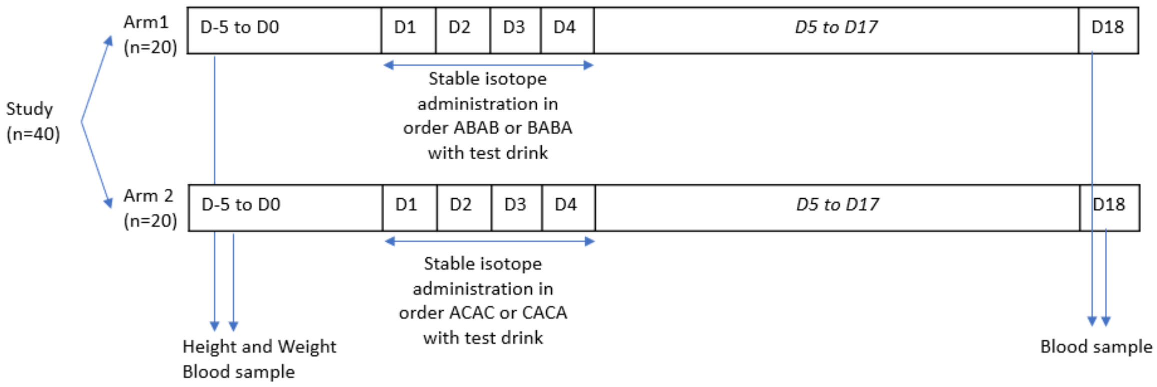

Figure S1. Study design. Stable isotopes were administered over 4 alternative day with the test drink (milk) i.e., A: ferrous sulfate labelled with ^{58}Fe, B: ferrous ammonium phosphate labelled with ^{57} Fe, C ferric pyrophosphate labelled with ^{57}Fe, after the subjects had fasted overnight.
